# Rituximab in the Treatment of Interstitial Lung Disease Associated with Autoimmune Diseases: Experience from a Single Referral Center and Literature Review

**DOI:** 10.3390/jcm9103070

**Published:** 2020-09-23

**Authors:** Belén Atienza-Mateo, Sara Remuzgo-Martínez, Diana Prieto-Peña, Víctor Manuel Mora Cuesta, David Iturbe-Fernández, Javier Llorca, Lara Sánchez-Bilbao, Alfonso Corrales, Gerardo Blanco Rodríguez, José Javier Gómez-Román, José Manuel Cifrián, Miguel Ángel González-Gay

**Affiliations:** 1 Research Group on Genetic Epidemiology and Atherosclerosis in Systemic Diseases and in Metabolic Bone Diseases of the Musculoskeletal System, IDIVAL, 39011 Santander, Spain; mateoatienzabelen@gmail.com (B.A.-M.); sara.r.mtz@gmail.com (S.R.-M.); diana.prieto.pena@gmail.com (D.P.-P.); victormanuel.mora@scsalud.es (V.M.M.C.); david.iturbe@scsalud.es (D.I.-F.); afcorralesm@hotmail.com (A.C.); josecifrian@gmail.com (J.M.C.); 2 ‘López Albo’ Post-Residency Programme, Hospital Universitario Marqués de Valdecilla, 39008 Santander, Spain; 3 Department of Rheumatology, Hospital Universitario Marqués de Valdecilla, 39008 Santander, Spain; lasanbil@gmail.com; 4 Department of Pneumology, Hospital Universitario Marqués de Valdecilla, 39008 Santander, Spain; 5 Department of Epidemiology and Computational Biology, School of Medicine, University of Cantabria, and CIBER Epidemiología y Salud Pública (CIBERESP), IDIVAL, 39011 Santander, Spain; javier.llorca@unican.es; 6 Department of Radiology, Hospital Universitario Marqués de Valdecilla, 39008 Santander, Spain; gerardo.blanco@scsalud.es; 7 Department of Pathology, Hospital Universitario Marqués de Valdecilla, 39008 Santander, Spain; josejavier.gomez@scsalud.es; 8 School of Medicine, Universidad de Cantabria, 39011 Santander, Spain; 9 Cardiovascular Pathophysiology and Genomics Research Unit, School of Physiology, Faculty of Health Sciences, University of the Witwatersrand, Johannesburg 2193, South Africa

**Keywords:** autoimmune diseases, interstitial lung disease, rituximab, review

## Abstract

In the present study, we aimed to report our experience with rituximab (RTX) in the treatment of patients with ILD associated with AD (AD-ILD) at a single center. For this purpose, clinical characteristics, radiological findings, and pulmonary function tests (PFTs) of RTX-treated AD-ILD-patients seen from May 2016 until March 2020 at a referral center for individuals with ILD were retrospectively reviewed. Additionally, an updated literature review was conducted. A total of 26 patients (mean age 58.3 ± 11.1 years at ILD diagnosis) was included. The most common ADs related to ILD were systemic sclerosis, idiopathic inflammatory myositis (including anti-synthetase syndrome) and rheumatoid arthritis. Non-specific interstitial pneumonia (n = 12) and usual interstitial pneumonia (n = 11) were the predominant radiological patterns. The sustained improvement in PFTs was observed from the start of RTX, with a statistically significant increase in DLCO from basal to one year after RTX (mean + 4.2%, *p* = 0.024). Overall, there were no differences when comparing PFT outcome according to the radiological pattern or the specific type of AD. In conclusion, RTX constitutes a good therapeutic option to preserve lung function in patients with AD-ILD, regardless of the radiological pattern or the underlying AD.

## 1. Introduction

Rheumatic autoimmune diseases (ADs) are a group of systemic conditions characterized by a dysregulation of the immune system [1,2]. Lung involvement in rheumatic ADs is not uncommon. It may be the result of parenchymal (airways or interstitium), pleural and/or vascular involvement. Interstitial lung disease (ILD) is one of the most frequent and serious pulmonary complications associated with ADs [3,4].

The prevalence and mortality of patients with AD-associated ILD (AD-ILD) varies depending on the specific AD and the ILD pattern. In this regard, systemic sclerosis (SSc) and idiopathic inflammatory myositis (IIM), especially anti-synthetase syndrome, are more frequently associated with ILD development, followed by rheumatoid arthritis (RA) [5,6,7]. While non-specific interstitial pneumonia (NSIP) is the histological and radiological ILD pattern more commonly associated to the majority of ADs, RA is usually related to a usual interstitial pneumonia (UIP) pattern, which implies a poorer prognosis [7,8,9,10].

The etiopathogenesis of AD-ILD remains unclear and the treatment of AD-ILD patients suppose a challenge for the clinicians. Nevertheless, the immune component of these conditions has been well-established, being the production of autoantibodies the cause or consequence of lung injury, lung inflammation and subsequent fibrosis [4,11]. Therefore, although there are no specific guidelines for the management of AD-ILD, immunosuppression is the major therapy target in these patients. Treatment choice is usually a shared decision between pneumologists and rheumatologists, based on personal experience, retrospective studies and case series. In this regard, several therapeutic options have yielded promising results, including lung transplantation as the last alternative [12,13]. Besides corticosteroids, cyclophosphamide and mycophenolate mofetil are the most widely used drugs among the conventional immunosuppressive drugs [14]. Rituximab (RTX), a chimeric (human/murine) monoclonal antibody against the surface antigen CD20, expressed on pre-B and B lymphocytes, has shown efficacy in the treatment of patients with AD-ILD, even as a rescue alternative in severe and refractory cases [15,16,17,18,19,20].

Most studies on RTX therapy are the result of collaboration among different centers [17,18,21,22,23,24,25,26,27]. In the present study, we aimed to report our experience with RTX in the treatment of patients with AD-ILD assessed at a single referral center for individuals with ILD.

## 2. Materials and Methods

### 2.1. Patients

We performed a retrospective study of consecutive patients with the diagnosis of AD-ILD followed at the ILD Unit of the Marqués de Valdecilla University Hospital (Santander, Spain) from May 2016 until March 2020. Patients were diagnosed and followed-up by the same group of clinicians. Given the proven RTX efficacy in several types of AD-ILD [15,16,17,18,19,20], we included patients with different underlying ADs. All patients had received RTX at some point during their disease course. The diagnosis of ILD was made by pneumologists, based on clinical and radiological findings and pulmonary function test (PFT) abnormalities, according to the clinical guidelines [8,28]. Histological confirmation was performed according to the pneumologist’s judgment. The diagnosis of the diverse rheumatic ADs was established before patients’ referral to the ILD Unit, or it was made with the collaboration of experienced rheumatologists during the ILD assessment. In most cases, RTX was prescribed as an off-label indication and, therefore, written informed consent was requested and obtained from all patients. In addition, informed consent for inclusion in the study was obtained from each patient. No competing clinical trials were conducted in the time interval covered by the study. The study was conducted in accordance with the Declaration of Helsinki, and the protocol was approved by the Ethics Committee of Clinical Research of Cantabria, Spain (2016.092).

### 2.2. Methods

Demographic and clinical features collected included ages at AD and ILD diagnosis, gender, type of AD, previous immunosuppressive treatment, lung high-resolution computed tomography (HRCT) interstitial pattern and PFTs. We also recorded data related to RTX administration, including age at RTX onset, number of cycles and dose administered, reason for starting RTX, discontinuation, adverse events and concomitant treatment.

To evaluate the efficacy of RTX, PFTs evolution was assessed at different points of the follow-up, when available: 1 year before starting RTX, close to the start of RTX (basal) and 6 months, 1 year and 2 years after the beginning of RTX therapy. Among the PFTs parameters, forced vital capacity (FVC), forced expiratory volume in the first second (FEV1), FEV1/FVC ratio and diffusing capacity of the lungs for carbon monoxide (DLCO) were recorded. Additionally, HRCT findings were collected at the most recent times before starting RTX and at the latest time after the last dose of RTX. The HRCT patterns were stratified according to the diagnostic criteria of UIP (Fleischner Society) [29].

Optimization, standing for a de-intensifying treatment strategy, was considered when the course of the disease was stable and the CD19 levels remained undetectable for at least 6 months after the last RTX infusion.

### 2.3. Literature Review

A comprehensive search of biomedical literature until May 2020 about the treatment of AD-ILD patients with RTX was performed. The research sources consulted were from MEDLINE, life science journals and online books published primarily on PubMed. We included cases reports to provide the maximum current evidence about this issue.

### 2.4. Statistical Analysis

Results were reported as the number of individuals (n) and percentage (%) for categorical variables. For continuous variables, the Shapiro–Wilk test was performed to determine the distribution of the data. Mean ± standard deviation (SD) or median (25th–75th interquartile range (IQR)) were used when data were normally or not normally distributed, respectively. For the comparison of PFTs percentages between two time points, paired Student’s t-test was used. This test was also performed in patients stratified according to UIP and NSIP patterns, and according to the most frequent underlying ADs (SSc, IIM and RA). To further compare PFTs values at multiple time points, we carried out a repeated-measures ANOVA test in the whole group of AD-ILD patients. Statistically significant differences were considered at *p* < 0.05. Statistical analysis was performed using the software STATA 12/SE (Stata Corp., College Station, TX, USA).

## 3. Results

### 3.1. Basal Data of the Patients

Twenty-six patients (13 women and 13 men; mean age at AD and ILD diagnosis of 55.5 ± 12.1 and 58.3 ± 11.1 years, respectively) from a cohort of 34 patients with AD-ILD, treated with RTX, were assessed in the present study. The remaining eight patients were excluded due to lung transplantation (n = 3), duration of follow-up at the time of evaluation less than 6 months (n = 2), lack of sufficient clinical information sent by the centers that referred patients (n = 2), and the intercurrence of a mantle lymphoma treated with RTX at hematological doses (n = 1). The removal of the three lung transplanted patients was due to the fact that they had only received a single RTX cycle before undergoing lung transplantation. The results of their PFTs after lung transplantation may have been influenced by the surgical procedure rather than the RTX treatment. Because of that, they could not be compared with the remaining patients undergoing RTX therapy from this series.

The types of AD related to ILD were grouped as follows: SSc (n = 7), IIM (n = 6; five with anti-synthetase syndrome and one with amyopathic dermatomyositis), RA (n = 5), interstitial pneumonia with autoimmune features (IPAF) (n = 3), primary Sjögren’s syndrome (n = 3) and myeloperoxidase anti-neutrophil cytoplasmic antibody (MPO-ANCA) positive (n = 2). Regarding HRCT findings, a NSIP pattern was found in 12 (46.2%) and an UIP pattern in 11 (42.4%) patients. The remaining three patients presented probable UIP, indeterminate for UIP and non-NSIP patterns; one each. Demographic and clinical characteristics of the AD-ILD patients are shown in Table 1.

The reasons for starting RTX were the existence of a clinically, radiologically, and/or functionally significant ILD (n = 12, 46.2%), the recognition of an AD in the course of an established ILD (n = 12, 46.2%) or the presence of active arthritis (n = 2; 7.6%). RTX was indicated as the first-line immunosuppressive option, combined with conventional immunosuppressive therapies in the majority of cases, in selected patients who presented an active and/or severe systemic affection.

### 3.2. Treatment Before Rituximab

Twenty patients (76.9%) had previously received glucocorticoids (n = 19; 73.1%) and/or conventional immunosuppressive treatment: methotrexate (n = 9; 34.6%), hydroxychloroquine (n= 9; 34.6%), azathioprine (n = 6; 23.1%), mycophenolate mofetil (n = 5; 19.3%), cyclophosphamide (n = 4; 15.4%), leflunomide (n = 3; 11.5%), sulphasalazine (n = 1; 3.8%) and tacrolimus (n = 1; 3.8%). In addition three patients with RA and another three patients who initially presented with predominant joint manifestations (23.1%) received previous biological therapy with abatacept (n = 3; 11.5%), etanercept (n = 3; 11.5%), adalimumab (n = 2; 7.7%), infliximab (n = 2; 7.7%), tocilizumab (n = 2; 7.7%) and/or golimumab (n = 1; 3.8%) to manage joint inflammation.

At the time of the indication for the administration of RTX, 19 patients were on immunosuppressive therapy, including glucocorticoids, hydroxychloroquine, mycophenolate mofetil, azathioprine and others (Table 1). In addition, two patients with SSc were receiving bosentan due to ischemic digital ulcers. Three patients were under antifibrotic therapy with nintedanib (n = 2; 7.7%) or pirfenidone (n = 1; 3.8%).

### 3.3. Rituximab and Concomitant Treatment

The mean age at RTX onset was 58.9 ± 10.2 years. The mean intervals between AD and ILD diagnosis and RTX initiation were 2.8 and 0.6 years, respectively. Dosing regimens of RTX infusion (previously administered i.v. methylprednisolone and dexchlorpheniramine) were 1000 mg i.v. on days 0 and 14 for 20 (76.9%) patients, 500 mg i.v. on days 0 and 14 for four patients (15.4%) or 375 mg/m^2^ i.v. once weekly × 4 doses for two patients (7.7%). The median number of RTX cycles was two (2.25–4.25).

Twenty-five patients (96.1%) received concomitant immunosuppressive treatment (Table 1). The concomitant treatment and dosages were the following: glucocorticoids (median dose of prednisone 6.25 (5–10) mg p.o./day), hydroxychloroquine (median dose 200 mg p.o./day), mycophenolate mofetil (median dose 2 g p.o./day), azathioprine (median dose 100 (50–100) mg p.o./day), sulphasalazine (1.5 g p.o./day), tacrolimus (2 g p.o./day), and immunoglobulins (a total of 2 g/kg. i.v. infused within 4 days). Among patients with SSc, another three required additional treatment with bosentan during follow-up due to severe Raynaud’s phenomenon with digital ulcers. Of the three patients on antifibrotic therapy, pirfenidone (n = 1) was discontinued, whereas nintedanib (n = 2) was maintained. However, during follow-up, one patient with nintedanib had to be switched to pirfenidone due to intense diarrhea.

### 3.4. Interstitial Lung Disease Status Preceding RTX Onset

There was a decline in PFTs at the time of the first RTX administration when compared with PFTs performed 1 year prior to RTX onset (Table 2). The mean FVC had fallen from 81.5% to 78.8%, the mean FEV1 had fallen from 79.0% to 78.6% and the mean DLCO decreased from 45.0% to 39.3%. In addition, two patients showed significant radiological progression of the ILD preceding RTX onset.

### 3.5. Outcome of AD-ILD Patients Undergoing RTX Treatment

**Figure 1:**
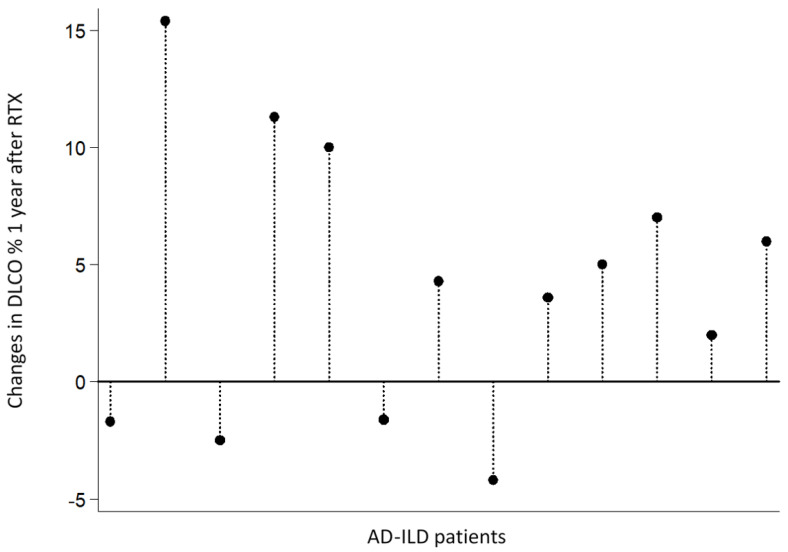
Changes in DLCO values in 13 AD-ILD patients 1 year after RTX. AD-ILD patients are ordered as follows: SSc-ILD (n = 4), IIM-ILD (n = 3), RA-ILD (n = 3), IPAF (n = 2) and MPO-ANCA-positive (n = 1). AD: autoimmune disease; DLCO: diffusing capacity of the lungs for carbon monoxide; ILD: interstitial lung disease; IIM: idiopathic inflammatory myopathies; IPAF: interstitial pneumonia with autoimmune features; MPO-ANCA: myeloperoxidase anti-neutrophil cytoplasmic antibody; RTX: rituximab; SSc: systemic sclerosis.

A sustained improvement of all lung functional parameters was observed from the start of RTX. In particular, an increase in mean FVC values (5.8% at 6 months, 0.5% at 1 year and 4.2% at 2 years), FEV1 (2.5% at 6 months and 3.4% at 2 years), and DLCO (0.4% at 1 year and 10.6% at 2 years) was disclosed (Table 2). Interestingly, we found a statistically significant increase in paired DLCO values (mean of differences + 4.2%, *p* = 0.024), comparing basal levels with those found one year after RTX (mean ± SD: 34.02 ± 14.75 vs. 38.22 ± 15.86, respectively). Figure 1 shows individual changes in DLCO values from 13 patients with available data both at RTX onset and 1 year after RTX. A significant increase in DLCO could be observed in 9 of these 13 patients after 1 year with RTX.

In addition, the repeated-measures ANOVA test performed in AD-ILD patients with available PFTs at basal RTX, 6 months and 1 year after RTX indicated a preserved pulmonary function, with no statistically significant differences (*p* values corrected for Greenhouse-Geisser epsilon = 0.11 for FVC, 0.38 for FEV1 and 0.21 for DLCO).

Chest HRCT after the last dose of RTX was available in 23 patients. There was a stabilization of interstitial lung abnormalities in 15/23 patients (65.2%), a worsening from baseline in 5/23 patients (21.7%) and a marked improvement in lung affection in 3/23 patients (13.1%).

### 3.6. PFTs Evolution According to UIP and NSIP Pattern

**Figure 2:**
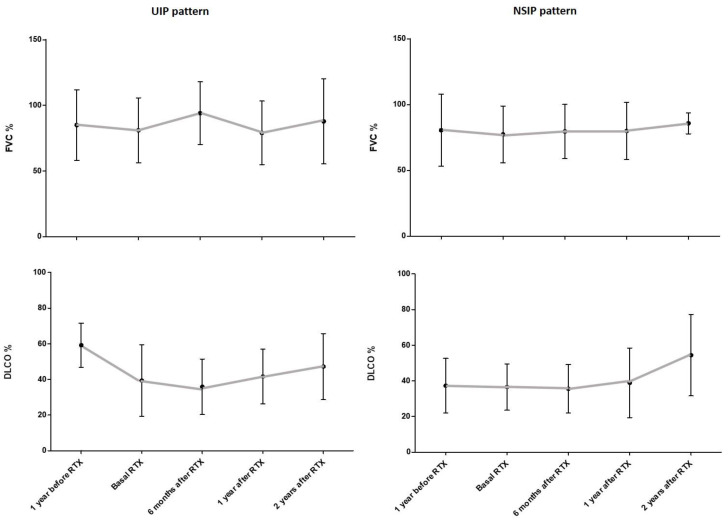
Evolution of FVC and DLCO values in AD-ILD patients included in this study according to their UIP and NSIP HRCT patterns. Bars indicate mean value and standard deviation for each time point. AD: autoimmune disease; DLCO: diffusing capacity of the lungs for carbon monoxide; FVC: forced vital capacity; HRCT: high-resolution computed tomography; ILD: interstitial lung disease; NSIP: non-specific interstitial pneumonia; RTX: rituximab; UIP: usual interstitial pneumonia.

A non-statistically significant decline in PFTs was observed when baseline data were compared with those from one year before RTX onset in patients stratified according to the radiological pattern, UIP or NSIP. In this regard, the mean FVC decreased from 85% to 81% for UIP and from 80.5% to 77.4% for NSIP, and the mean DLCO decreased from 59.2% to 39.4% for UIP and from 37.3% to 36.5% for NSIP. All lung functional parameters improved at every time of assessment, regardless of the radiological pattern. At 2 years of follow-up with RTX treatment, the mean FVC was 87.8% for UIP and 85.8% for NSIP, and the mean DLCO was 47.2% for UIP and 54.4% for NSIP. The evolution of FVC and DLCO values in AD-ILD patients, according to the UIP and NSIP patterns, is shown in Figure 2.

### 3.7. PFTs Evolution According to the Underlying AD

**Figure 3:**
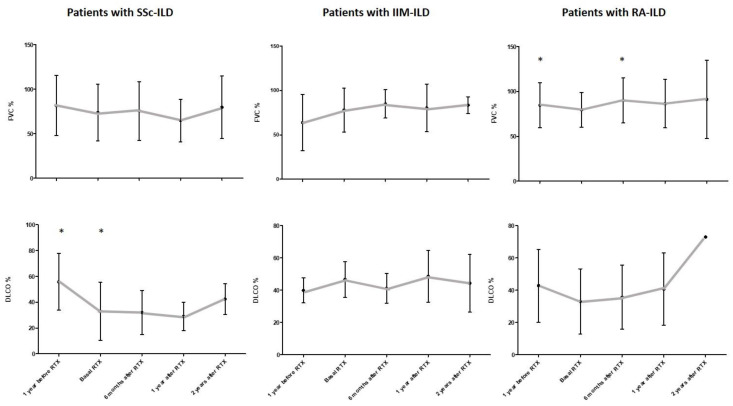
Evolution of FVC and DLCO values in SSc-ILD, IIM-ILD and RA-ILD patients included in this study. Bars indicate mean value and standard deviation for each time point. * indicates statistically significant differences. AD: autoimmune disease; DLCO: diffusing capacity of the lungs for carbon monoxide; FVC: forced vital capacity; IIM: idiopathic inflammatory myositis; ILD: interstitial lung disease; RA, rheumatoid arthritis; RTX, rituximab; SSc, systemic sclerosis.

Regarding the specific type of AD associated with ILD, all groups showed a stability of the PFTs from the beginning of RTX treatment. Importantly, there was a statistically significant decrease in DLCO levels in SSc-ILD patients after the analysis of paired DLCO values from the year before RTX to the time of RTX onset (mean ± SD: 51.50 ± 24.74 vs. 45.87 ± 26.24, respectively; mean of differences −5.63%, *p* = 0.029). In addition, there was a statistically significant improvement in mean FVC in RA-ILD patients when comparing by pairs the year before RTX and 6 months after RTX onset (mean ± SD: 84.60 ± 25.08 vs. 93.43 ± 24.54, respectively; mean of differences +8.83%, *p* = 0.022). Figure 3 shows the evolution of FVC and DLCO values in the most representative groups of AD-ILD patients (SSc, IIM and RA).

### 3.8. Discontinuation, Adverse Events and Optimization of Rituximab

RTX was discontinued in six patients (23.1%) due to pulmonary embolism, recurrent respiratory infections, lung cancer, diverticulitis complicated with infection of the surgical wound, uncontrolled arthritis and inefficacy; one each. The appearance of a cutaneous rash after RTX administration in one patient and an infusion reaction in another patient were reported as adverse events. During the follow-up, three patients (11.5%) died, two because of lower respiratory tract infection and one due to lung cancer. All of them had previously withdrawn RTX and were on another therapy at the time of death.

Treatment with RTX was optimized in 12 patients (46.1%) following different strategies: increasing the administration interval by more than 6 months (n = 5, 19.2%), reducing half the dose to 500 mg (n = 4, 15.4%) or combining both regimens (n = 3, 11.5%), according to each individualized case.

## 4. Discussion

This retrospective study presents the experience with RTX treatment in AD-ILD patients from a single ILD center. The results suggest that timely administered RTX may help preserve pulmonary function in patients with AD-ILD for at least 2 years.

Nowadays, there is a growing awareness of the significant morbidity and mortality that the presence of an AD-ILD entails [5,6,7]. In this sense, several reports have been published on the diagnostic and therapeutic management of this group of diseases [12,14,30]. Most of these reports indicate the use of mycophenolate mofetil and cyclophosphamide as the main conventional immunosuppressive drugs for the treatment of patients with AD-ILD. Azathioprine, cyclosporine and tacrolimus have also been reported as reasonable options for these patients. However, conventional immunosuppressive therapy is not always enough to control lung affection or is contraindicated.

Recently, RTX has shown efficacy in the treatment of patients with AD-ILD [19]. This biological drug leads to B-cell peripheral depletion and, consequently, secondary inflammation inhibition [31]. RTX has been approved by the EMA and FDA for the treatment of lymphoproliferative syndromes, rheumatoid arthritis, ANCA-associated vasculitis and pemphigus vulgaris. Moreover, RTX treatment may be considered in organ-threatening, refractory systemic lupus erythematosus [32] and in refractory severe extra-glandular manifestations in patients with primary Sjögren’s syndrome [33,34].

The evidence of the use and safety of RTX in AD-ILD patients varies according to the considered AD. Several studies have shown the efficacy of RTX in IIM, particularly in anti-synthetase syndrome, and SSc-related ILD [21,22,23,24,35,36,37]. The results of these reports show, in general, an improvement in PFTs in IIM-ILD patients and a stabilization of lung affection in SSc-ILD patients treated with RTX. Experience with RTX in other AD with lung involvement is scarce [16,38,39,40,41]. Our series also included 2 MPO-ANCA-positive patients with pulmonary fibrosis but no other manifestations of systemic vasculitis. This group of patients might be called “pulmonary limited vasculitis” as a phenotypic variant of microscopic polyangiitis, as stated by Katsumata et al. [42]. There is no reported evidence about RTX treatment in this subtype of patients. Regarding safety, it is well known that the most common adverse events in patients with RTX treatment are infusion reactions and mild to severe infections [16,17,18,24,43], similar to our findings.

Although the natural history of pulmonary function in patients with AD-ILD is characterized in progressive fibrosing phenotypes by a significant decline in FVC and/or DLCO values over time, an increase in lung HRCT extension, and a worsening of symptoms, leading to a high mortality [44], it is well known that, in many cases, AD-ILD is not progressive. In our study all patients were treated early in the disease course (after ILD diagnosis), which restricted the possibility to define the progressive phenotypes in our population. In this regard, it is noteworthy that, in contrast to other studies where RTX was initiated when severe ILD was established [15,16,20], in the present study of 26 patients with AD-ILD, pulmonary affection was mild, with mean FVC and FEV1 > 70% at the time of RTX onset. Mean values of FVC and FEV1 remained over 70% in every stage of assessment until 2 years of follow-up, and mean DLCO levels increased from severe to moderate. Furthermore, the majority of patients achieved a radiological ILD stabilization or improvement, and patients with HRCT images of progression did not have clinical repercussions. This was in line with previous similarly designed studies [20,45]. Consequently, this management may have some influence in the natural history/ILD progression in AD-ILD patients. Nevertheless, this assumption should be confirmed with the presence of an untreated group to compare longitudinal changes in physiology.

The concept of minimal clinically important difference (MCID) for FVC has been assessed in ILD patients [46,47]. In this regard, du Bois et al. estimated that the MCID for percent predicted FVC was between 2–6% in a large cohort of patients with idiopathic pulmonary fibrosis [46]. Furthermore, Patel et al. reported a similar MCID range for FVC in patients with ILD, including, for the first time, an AD-ILD group [47]. Although the differences in mean FVC values could be assumed to be clinically important in our study, given its design, this issue should be interpreted with caution.

A comprehensive updated literature review about the outcome of lung function and HRCT of patients with AD-ILD treated with rituximab is shown in Table 3. Former retrospective cohorts’ studies of RTX treatment in heterogeneous AD-ILD groups, like ours, have described an acceptable evolution of PFTs and lung HRCT patterns [16,17,18,20,45,48]. Our series showed an FVC stabilization during 24 months of follow-up, which was in line with the results of Lepri et al. [17], whereas a relevant increase in FVC at 6–12 months was reported by other authors [16,18,20,45]. Interestingly, a significant improvement in DLCO at 12 months was observed in our study. This finding was in keeping with Robles-Perez et al. findings. In this regard, these authors reported an improvement of DLCO after 12 and 24 months of RTX treatment [20]. As reported by Chartrand et al. [48], we found no differences in PFTs evolution throughout the follow-up, according to UIP or NSIP HRCT patterns. In contrast, Duarte et al. and Robles-Perez et al. reported a better outcome of NSIP pattern [18,20].

Our study has potential limitations to be considered. Firstly, its retrospective nature did not allow us to establish with certainty whether the changes observed in PFTs from RTX start represented an MCID associated with the improvement of the quality of life or symptoms of the patients. Furthermore, some PFTs values were missing or incomplete at different points of the follow-up. Next, regarding treatment, the dosing of immunosuppressive medication with RTX was not uniform since it depended on the treating physician judgment as the study was based on real life clinical practice. Moreover, in some patients, concomitant immunosuppressive therapy was initiated at the start of RTX therapy. Therefore, management was more intensive than before RTX onset, due to the simultaneous initiation of RTX with additional conventional immunosuppressive treatment. Because of that, we could not precisely exclude that the stabilization or improvement in their PFTs could have been influenced not only by the effect of RTX but also by concomitant immunosuppressive therapy. Despite all these considerations, our results indicate that the course of lung disease did not worsen in our patients. As a whole, our results are in line with the majority of previous reports, supporting the claim that RTX is a promising therapy useful to maintain or improve lung function in AD-ILD patients. Moreover, in accordance with previous evidence, the optimization of RTX was possible in selected cases of our study, with no detrimental effects.

In conclusion, RTX constitutes a good therapeutic option to preserve lung function in patients with AD-ILD, regardless of the radiological pattern or the underlying AD. Furthermore, RTX optimization is possible and safe.

## Figures and Tables

**Table 1:** Demographic and clinical characteristics of 26 AD-ILD patients included in this study.

Characteristics	
Sex (Women/Men), n (%)	13/13 (50.0/50.0)
Age at AD diagnosis, years, mean ± SD	55.5 ± 12.1
Age at ILD diagnosis, years, mean ± SD	58.3 ± 11.1
Age at RTX onset, years, mean ± SD	58.9 ± 10.2
Rheumatic autoimmune disease, n (%)	
Systemic sclerosis	7 (26.9)
Idiopathic inflammatory myositis	6 (23.1)
Rheumatoid arthritis	5 (19.3)
Interstitial pneumonia with autoimmune features	3 (11.5)
Primary Sjögren’s syndrome	3 (11.5)
MPO-ANCA positive	2 (7.7)
High-resolution computed tomography pattern, n (%)	
UIP pattern	11 (42.4)
Probable UIP pattern	1 (3.8)
Indeterminate for UIP pattern	1 (3.8)
Features most consistent with an alternative diagnosis	
NSIP pattern	12 (46.2)
Non-NSIP pattern	1 (3.8)
IS treatment at RTX indication, n (%)	
Glucocorticoids	18 (69.2)
Hydroxychloroquine	5 (19.3)
Mycophenolate mofetil	4 (15.4)
Azathioprine	3 (11.5)
Methotrexate	1 (3.8)
Sulphasalazine	1 (3.8)
Tacrolimus	1 (3.8)
Tocilizumab	1 (3.8)
Abatacept	1 (3.8)
Concomitant treatment, n (%)	
Glucocorticoids	21 (80.7)
Hydroxychloroquine	8 (30.7)
Mycophenolate mofetil	7 (26.9)
Azathioprine	3 (11.5)
Sulphasalazine	1 (3.8)
Tacrolimus	1 (3.8)
I.V. immunoglobulins	1 (3.8)

AD: autoimmune disease; ILD: interstitial lung disease; IS: immunosuppressive; NSIP: non-specific interstitial pneumonia; RTX: rituximab; SD: standard deviation; UIP: usual interstitial pneumonia.

**Table 2:** Comparison of pulmonary function tests in 26 AD-ILD patients included in this study.

	1 Year before RTX	Basal RTX	6 Months after RTX	1 Year after RTX	2 Years after RTX
**FVC %, mean ± SD**	81.5 ± 26.7	78.8 ± 22.7	84.6 ± 22.9	79.3 ± 22.5	83.0 ± 26.2
**FEV1 %, mean ± SD**	79.0 ± 22.7	78.6 ± 22.2	81.1 ± 21.3	78.6 ± 21.5	82.0 ± 24.9
**DLCO %, mean ± SD**	45.0 ± 16.3	39.3 ± 15.8 *	37.1 ± 13.7	39.7 ± 16.2 *	49.9 ± 18.5

AD: autoimmune diseases; DLCO: diffusing capacity of the lung for carbon monoxide; FEV1: forced expiratory volume in one second; FVC: forced vital capacity; ILD: interstitial lung disease; RTX: rituximab; SD: standard deviation. * *p* = 0.024.

**Table 3:** Literature review of patients with AD-ILD treated with rituximab.

Author ^a^	Study Design	Number of Cases ^b^	Rheumatic ADs Included (n)	Follow-Up (Months) ^c^/Cycles	ILD Improvement
McGonagle et al. [49]	Case report	1	SSc	24/2	FVC, DLCO and HRCT
Yáñez et al. [50]	Case report	1	IIM (DM)	15/2	FVC, FEV1, DLCO and HRCT
Lafyatis et al. [51]	Clinical trial	7	SSc	6/1	(FVC, DLCO and HRCT stb)
Sem et al. [52]	Retrospective cohort	11	IIM (ASS)	6/1	FVC (n = 6) and DLCO (n = 3) HRCT (n = 5/9)
Vandenbroucke et al. [53]	Case report	1	IIM (ASS)	3/1	FVC, FEV1 and HRCT
Daoussis et al. [54]	Clinical trial	8	SSc	12/2	FVC and DLCO (HRTC stb)
Yoo [55]	Case report	1	SSc	1/1	FVC, DLCO and HRCT
Haroon et al. [56]	Case report	1	SSc	12/1	FVC, FEV1, DLCO and HRCT
Zappa et al. [57]	Case report	1	IIM (ASS)	14/2	FVC, FEV1, DLCO and HRCT
Hartung et al. [58]	Case report	1	RA	8/2	FVC (no HRCT performed)
Marie et al. [59]	Retrospective cohort	7	IIM (ASS)	12/2	FVC, FEV1 and DLCO HRCT (n = 5; +2 stb)
Keir et al. [15]	Retrospective case series	8	IIM (ASS) (4) Undifferentiated CTD (2) IIM (DM) (1) SSc (1)	9–12/ND	FVC and DLCO (n = 5) Symptoms
Daoussis et al. [60]	Clinical trial	8	SSc	24/4	FVC and DLCO/HRCT (n = 5)
Keir et al. [16]	Retrospective cohort	33	IIM (10) Undifferentiated CTD (9) SSc (8) RA (2) Mixed CTD (2) SLE (1) SS (1)	12/ND	FVC and DLCO (n = 10) (+18 stb)
Unger et al. [61]	Retrospective cohort	11	IIM (ASS)	30/1–3	FVC TLC (n = 6/8) and DLCO (n = 6/6)
Jordan et al. [24]	Observational case control	9	SSc	6 (4–12)/ND	DLCO (FVC stb)
Sumida et al. [62]	Case report	1	SSc overlap SLE	>6/2	FVC, DLCO and HRCT
Bosello et al. [63]	Clinical trial	14	SSc	48.5 (20.4)/ND	FVC (n = 3, + 10 stb), DLCO (n = 4, + 7 stb), HRCT (n = 2, + 10 stb)
Andersson et al. [35]	Retrospective cohort	24	IIM (ASS)	52 (11–118)/ 2.7 (1–11)	FVC and DLCO HRCT (n = 22/23)
Koichi et al. [64]	Case report	1	IIM (amyopathic DM)	2/1	HRCT (no PFT performance)
Watanabe et al. [65]	Case report	1	IIM (amyopathic DM)	6/1	HRCT (FVC and FEV1 stb)
Giuggioli et al. [25]	Retrospective cohort	8	SSc	37 (21)/1–5	(FVC, DLCO and HRCT stb n = 6)
Fitzgerald et al. [43]	Retrospective cohort	10	SSc (6) RA (4)	12.3 (3–27)/ND	FVC (n = 7) and DLCO (n = 7) HRCT (n = 6/7)
Allenbach et al. [21]	Clinical trial	10	IIM (ASS)	12/2	n.s. FVC (n = 4) and DLCO (n = 2) HRCT (n = 1; + 8 stb)
Chartrand et al. [48]	Retrospective cohort	24	RA (15) IIM (3) IIM overlap RA (2) SSc (3) Suggestive CTD/IPAF (1)	35.6 (19.3)/ND	FVC (in 8/14 multiple cycles) (FVC and HRCT stb in all patients at 6 months)
Dasa et al. [66]	Case report	1	IIM (ASS)	12/2	FVC, FEV1, DLCO and HRCT
Paola et al. [67]	Case report	1	CTD anti-SS-A/Ro-52 positive	ND/1	FVC and DLCO
Sharp et al. [45]	Retrospective cohort	24	IIM (ASS) (10) Unclassifiable CTD-ILD (4) IIM other than ASS (3) SSc (3) SS (2) SLE (2)	29.6 (16.7)/ND	FVC (>10% in four) (DLCO stb) (FVC and DLCO changes greater in IIM subgroup) (HRCT improvement/stb in 13/22)
Eissa et al. [68]	Case report	1	IIM (DM)	>6/2	FVC, DLCO and HRCT
Bauhammer et al. [69]	Retrospective cohort	11	IIM (ASS)	35/4.6	FVC and DLCO
Lepri et al. [17]	Retrospective cohort	44	SSc (23) IIM (ASS) (15) Mixed CTD (6)	24/ND	FVC and DLCO stb (n.s. improvement) (ASS greater response)
Chen et al. [39]	Retrospective cohort	10	SS	26.1 (9–60)/1–5	DLCO and symptoms (FVC and HRCT stb)
Daoussis et al. [22]	Clinical trial	33	SSc	48 (12–84)/>2	FVC and DLCO
Ebata et al. [70]	Case report	1	SSc	23/2	FVC, DLCO and HRCT
Boonstra et al. [71]	Clinical trial	8	SSc	24/2	HRCT in 2/7 (4/7 stb) (FVC and DLCO stb)
Md Yusof et al. [40]	Prospective observational cohort	56	RA	6–12/ND	FVC and DLCO in 7/37 (stb in 25/37) HRCT in 1/14 (6 stb)
Chao et al. [72]	Case report	1	IIM (ASS)	5/1	FVC, DLCO and HRCT
Mohammed et al. [73]	Retrospective case series	4	SSc	6 months after the 4th cycle	FVC, FEV1 and DLCO (in 3/3) HRCT in 1/4 (stb in 2)
Sari et al. [37]	Retrospective cohort	14	SSc	15 (6–24)/1–5	FVC stb (>10% in four) (HRCT stb in 7/10)
Numajiri et al. [74]	Case report	1	SSc	22/2	FVC, DLCO and HRCT
Doyle et al. [23]	Retrospective cohort	25	IIM (ASS)	36/≥1	(FVC and DLCO stb, significant FVC improvement at 3 years in 7/7) (HRCT stb)
Thiebaut et al. [75]	Retrospective case control	7	SSc	24 (12–46)/1–6	(FVC and DLCO stb) Greater response than controls
So et al. [76]	Retrospective case series	4	IIM (amyopathic DM)	6–24/1	FVC and DLCO HRCT in 3/4 (1 stb)
Fraticelli et al. [77]	Prospective cohort	15	SSc	12/2	FVC, FEV1 and HRCT (DLCO stb)
Sircar et al. [36]	Clinical trial	30	SSc	6/2	FVC
Jensen et al. [78]	Retrospective case series	4	IIM (ASS)	84 (12–132)/≥1	FVC, DLCO and HRCT with RTX as induction therapy (2/4)
Melsens et al. [26]	Clinical trial	10	SSc	24/2	(FVC and DLCO stb)
Duarte et al. [18]	Retrospective cohort	49	RA (30) SSc (5) IIM (5) SLE (5) SS (4)	36 (12–72)/2 (1–4)	FVC at 12 months (DLCO stb)
Elhai et al. [27]	Prospective cohort	146	SSc	24.3 (13–41)/≥1	(FVC, DLCO and HCRT stb)
Rüegg et al. [79]	Case report	1	IIM (ASS)	18/3	FVC, DLCO and HRCT
Kourkouni et al. [80]	Case report	1	IIM (ASS)	12/2	FVC, DLCO and HRCT
Melissaropoulos et al. [81]	Case report	1	SSc	36/6	FVC, DLCO and HRCT
Ebata et al. [82]	Retrospective cohort	9	SSc	24/3	FVC and DLCO
Duarte et al. [83]	Retrospective cohort	26	RA	36/4 (1–12)	HRCT in three (stb in 9) (FVC stb)
Vadillo et al. [41]	Prospective observational cohort	31	RA	132 maximum/ 3.4 (1–18)	Higher probability of remaining free of functional impairment ^d^
Robles-Perez et al. [20]	Retrospective cohort	18	SSc (7) RA (5) SLE (4) IIM (ASS) (1) SS (1)	24/2–6	FVC and DLCO at 12 months DLCO at 24 months (Stb or improvement in 10/13 HRCT at 12 months)
Atienza-Mateo et al.; present series	Retrospective cohort	26	SSc (7) IIM (6) RA (5) IPAF (3) SS (3) MPO-ANCA positive (2)	24/2 (2.25–4.25)	DLCO in nine at 12 months HRCT in 3/23 (HRCT stb in 15/23) (FVC and FEV1 stb)

AD: autoimmune disease; ASS: anti-synthetase syndrome; CTD: connective tissue disease; DLCO: diffusing capacity of the lung for carbon monoxide; DM: dermatomyositis; FEV1: forced expiratory volume in one second; FVC: forced vital capacity; HRCT: high-resolution computed tomography; IIM: idiopathic inflammatory myopathy; ILD: interstitial lung disease; IPAF: interstitial pneumonia with autoimmune features; MPO-ANCA: myeloperoxidase anti-neutrophil cytoplasmic antibody; ND: non data; n.s.: non-statistically significant; RA: rheumatoid arthritis; SLE: systemic lupus erythematosus; SS: Sjögren’s Syndrome; SSc: Systemic Sclerosis; stb: stabilization. ^a^ Order of authors following ascending electronic publication date. ^b^ Cases defined as analyzed patients with AD-ILD treated with RTX. ^c^ Follow-up from the first RTX cycle administration. Data expressed as n, n (SD) or n (25th–75th IQR). ^d^ Compared with other therapies.
